# Inhibition of release of inflammatory mediators in primary and cultured cells by a Chinese herbal medicine formula for allergic rhinitis

**DOI:** 10.1186/1749-8546-2-2

**Published:** 2007-02-15

**Authors:** George B Lenon, Charlie CL Xue, David F Story, Frank CK Thien, Sarah McPhee, Chun G Li

**Affiliations:** 1 The RMIT Chinese Medicine Research Group, RMIT University, Plenty Road, Bundoora 3083, Australia; 2 The Natural Products Research Group, School of Medical Sciences, RMIT University, Plenty Road, Bundoora 3083, Australia; 3 Alfred Hospital & Monash University, Commercial Road, Melbourne 3004, Australia

## Abstract

**Background:**

We demonstrated that a Chinese herbal formula, which we refer to as RCM-101, developed from a traditional Chinese medicine formula, reduced nasal and non-nasal symptoms of seasonal allergic rhinitis (SAR). The present study in primary and cultured cells was undertaken to investigate the effects of RCM-101 on the production/release of inflammatory mediators known to be involved in SAR.

**Methods:**

Compound 48/80-induced histamine release was studied in rat peritoneal mast cells. Production of leukotriene B_4 _induced by the calcium ionophore A23187 was studied in porcine neutrophils using an HPLC assay and lipopolysaccharide-stimulated prostaglandin E_2 _production was studied in murine macrophage (Raw 264.7) cells by immune-enzyme assay. Expression of cyclooxygenase-1 (COX-1) and cyclooxygenase-2 (COX-2) was determined in Raw 264.7 cells, using western blotting techniques.

**Results:**

RCM-101 (1–100 μg/mL) produced concentration-dependent inhibition of compound 48/80-induced histamine release from rat peritoneal mast cells and of lipopolysaccharide-stimulated prostaglandin E_2 _release from Raw 264.7 cells. Over the range 1 – 10 μg/mL, it inhibited A23187-induced leukotriene B_4 _production in porcine neutrophils. In addition, RCM-101 (100 μg/mL) inhibited the expression of COX-2 protein but did not affect that of COX-1.

**Conclusion:**

The findings indicate that RCM-101 inhibits the release and/or synthesis of histamine, leukotriene B_4 _and prostaglandin E_2 _in cultured cells. These interactions of RCM-101 with multiple inflammatory mediators are likely to be related to its ability to reduce symptoms of allergic rhinitis.

## Background

Allergic rhinitis, in particular seasonal allergic rhinitis (SAR) or hay fever, is a common allergic condition [1]. World-wide, SAR afflicts 10 – 40% of individuals [2], with approximately 20% affected in the United States, 13% in Western Europe [3] and 16.1% in Australia [4]. SAR is an immune response to a wide variety of pollens from grasses, weeds and trees. It involves the interaction of allergens with specific immunoglobulin E (IgE) antibodies bound to high affinity Fcε receptors on the surface of mast cells and basophils in the nasal mucosa [5]. This interaction causes degranulation of these cells, releasing a number of inflammatory mediators which are responsible for a cascade of symptoms. Histamine, tryptase, prostaglandin and bradykinin are responsible for the immediate allergic response of sneezing, nasal itch and rhinorrhoea [5]. The late phase response, usually 4 – 6 hours after the immediate response, involves a large increase of eosinophils, basophils and other leukocytes at the inflammatory sites, in response to chemoattractants. In the late phase response, it is likely that histamine and leukotrienes are released from basophils rather than from mast cells because there is no corresponding increase in tryptase which originates from mast cells [5].

The conventional management of SAR is usually symptomatic, with histamine H_1 _receptor antagonists, sympathomimetic amine vasoconstrictors and corticosteroids. However, these treatments frequently have certain undesirable side effects and, often do not provide complete symptom relief [6]. Except corticosteroids, which have more significant side-effects, conventional treatments usually target a single inflammatory mediator, which probably explains their limited effectiveness [7].

Complementary/alternative therapies are becoming increasingly used in Western countries for the treatment of allergic diseases, with growing perceptions that such treatments are effective and that they are associated with fewer and less severe side effects [8]. Certain Chinese herbal formulae have been reported to be beneficial for the treatment of asthma and allergic rhinitis, including SAR, with some results showing that their effectiveness is comparable to prednisolone [8]. Recently, we conducted a randomized placebo-controlled clinical trial on a Chinese herbal formula which was developed from a traditional Chinese medicine formula for the treatment of symptoms associated with rhinitis. The formula was optimized on the basis of Chinese medicine syndrome theory for the treatment of SAR. We demonstrated that, after eight weeks of treatment, the herbal medicine formula, which we refer to as RCM-101, was effective in reducing the nasal and non-nasal symptoms of SAR [9].

In a previous investigation of the possible mechanism(s) of the anti-inflammatory/anti-allergic activity of RCM-101 in SAR, we found that the herbal formula inhibited histamine release from isolated guinea-pig tracheal preparations and the production of nitric oxide and prostaglandin E_2 _by cultured macrophages [10]. In the present study, as an extended investigation into the pharmacological activities of RCM-101 in reducing the symptoms of SAR, we have investigated its effects on histamine release, leukotriene B_4 _(LTB_4_) and prostaglandin E_2 _(PGE_2_) production, and the expression of two enzymes involved in inflammatory processes, namely cyclooxygenase-1 (COX-1) and cyclooxygenase-2 (COX-2).

## Methods

All experimental procedures involving animals were approved by RMIT University Animal Ethics Committee and were conducted in compliance with the Australian National Health and Medical Research Council guidelines.

Histamine, LTB_4 _and PGE_2 _are three key inflammatory mediators in allergic conditions such as SAR. To investigate the effects of RCM-101 on the synthesis/release of these mediators, we used three well characterized cell-based models, namely rat peritoneal mast cells for histamine [11], porcine neutrophils for LTB_4 _[12] and murine macrophage cells (Raw 164.7) for PGE_2 _[13,14].

### Preparation and extraction of RCM-101

RCM-101 is a herbal formula with 18 herbal ingredients, modified from a traditional Chinese medicine formula. Each herb for the formula was supplied in a granulated form produced under Good Manufacturing Practices by Min Tong Pharmaceutical Company (Taichong, Taiwan) which holds certification from the Australian Therapeutic Goods Administration (TGA-GMP No: 1888). Authenticated, quality-certified raw herbs were first tested to ensure that they were free of heavy metals. They were then washed, dried and extracted in boiling water for 1 – 1.5 hour. The aqueous extract was separated by filtration (100 mesh) and the water content was reduced to 60% by heating (50 – 60°C) under reduced pressure (50 – 70 mmHg) for 2 – 5 hours. The concentrated extract of each herb was combined with starch as an excipient and the product was dried and ground into fine granules. For each preparation, 1 g of granulated product was equivalent to 5 g of the raw herb. The granulated herbal preparations were sterilised and sealed in plastic bottles. In our laboratory, the granulated preparations of the herbs were combined in the proportions given in Table 1 to produce the herbal formula. All herbal ingredients of RCM-101 are approved in the Australian Register of Therapeutic Goods as active raw herbs for use in medicines.

**Table 1:** Herbal constituents of RCM-101 (% of granulated herbs by weight*)

**Scientific name**	**Botanical name**	**Chinese name**	**%**
*Flos Magnoliae*	*Magnolia liliflora *(Desr.)	*Xin Yi*	3.81
*Frutus Schisandrae Chinensis*	*Schisandra Chinensis *(Turcz.)	*Wu Wei Zi*	2.25
*Frutus Terminaliae Chebulae*	*Terminalia chebula *Retz.	*He Zi*	13.87
*Frutus Xanthii Sibirici*	*Xanthii Sibirici *Patr. Ex Widd.	*Cang Er Zi*	7.11
*Herba Asari*	*Asarum sieboldii *Miq.	*Xi Xin*	3.81
*Herba Menthae Haplocalysis*	*Mentha haplocalyx *Briq.	*Bo He*	4.68
*Herba Schizonepetae Tenuifoliae*	*Schizonepeta Tenuifolia *Briq.	*Jing Jie*	14.21
*Pericappium Citri Reticulatae*	*Citrus reticulata *Blanco	*Chen Pi*	9.36
*Radix Angelicae Sinensis*	*Angelica sinensis *(Oliv.) Diels	*Dang Gui*	4.68
*Radix Astragali Membranaceus*	*Astragalus membranaceus *(Fisch.) Bge	*Huang Qi*	4.68
*Radix Bupleuri*	*Bupleurum chinense *D.C	*Chai Hu*	3.81
*Radix Codonopsitis pilosulae*	*Codonopsis pilosula *(Franch.) Nannf.	*Dang Shen*	2.25
*Radix Glycyrrhizae Uralensis*	*Glycyrrhiza uralensis *(Fisch.)	*Gan Cao*	4.68
*Radix Saposhnikoviae Divaricata*	*Saposhnikovia divaricata *(Turcz.)	*Fang Feng*	4.51
*Rhizoma Atractylodis Macrocephalae*	*Atractylodes macrocephala *Koidz	*Bai Zhu*	4.68
*Rhizoma Cimicifugae*	*Cimicifuga foetida *L.	*Sheng Ma*	4.68
*Rhizoma Ligustici Chuanxiong*	*Ligusticum chuanxiong *(Hort.)	*Chuan Xiong*	4.68
*Semen Plantaginis*	*Plantago asiatica *L. Wild.	*Che Qian Zi*	2.25

* 1 g of each granulated herb is equivalent to 5 g of the raw herb (dry weight).

The herbal formula was extracted with ethanol (120 mg/mL) at room temperature with continuous agitation for 4 hours. The ethanol extract was collected by centrifugation (5000 rpm for 10 minutes) and vacuum filtration. The extract was dried using a rotary evaporator (Büchi Rotavapor, Brinkman Company, Westbury, NY, USA) and stored below -20°C. It was diluted to the required concentrations on the day of use.

### Reagents

Compound 48/80, histamine hydrochloride, O-phthalaldehide, spermidine hydrochloride, bovine serum albumin (BSA), phosphate buffer saline, heparin, disodium ethylenediaminetetraacetic acid (EDTA), lipopolysaccharide (LPS) *E.Coli*, calcium ionophore A23187, Hanks' balanced salt solution, RPMI 1640 medium, fetal bovine serum, phenylmethylsulfonyl fluoride, gentamycin, leupeptin, pepstatin A and nordihydroguaiaretic acid (NDGA) were obtained from Sigma Chemical Company (St Louis, MO, USA). Monoclonal mouse anti-rat cyclooxygenase 2 antibodies and mouse macrophage lysate were obtained from Transduction Laboratories (Lexington, KY, USA). Goat anti-rabbit horseradish peroxidase conjugated immunoglobulin G was obtained from Dako Corporation (CA, USA). The Coomassie blue protein assay kit was purchased from Bio-Rad (USA). Polyclonal rabbit anti-mouse cyclooxygenase-1 antibody, immune-enzyme analysis PGE_2 _kit, arachidonic acid, prostaglandin B_2_, LTB_4_, 6-trans LTB_4_, 6 trans-12 epi LTB_4_, 5-hydroxyeicosatetraenoic acid (5-HETE) and 15-hydroxyeicosatetraenoic acid (15-HETE) were obtained from Cayman Chemical Company (Ann Arbor, MI, USA). HPLC-grade methanol was supplied by Selby-Biolab (Clayton, Victoria, Australia). All other analytical reagents were obtained from Merck Pty Ltd (Kilsyth, Victoria, Australia).

### Histamine release from rat peritoneal mast cells

Rat peritoneal mast cells were collected in Tyrode buffer as previously described [11]. Briefly, rats (Sprague-Dawley, 200 – 300 g) of either sex were killed and 10 mL of Tyrode buffer (NaCl, 137 mM; KCl, 2.7 mM; HEPES, 10 mM; MgCl_2_, 1 mM; CaCl_2_, 1.0 mM; NaH_2_PO_4_, 0.41 mM), containing 0.3% BSA and 5 units/mL heparin was injected into the peritoneal cavity. The abdomen was gently massaged for about 90 seconds, and then carefully opened and the cell-containing peritoneal fluid collected with a transfer pipette. The cell-containing fluid was centrifuged at 4°C at 800 rpm for 5 minutes. The cells were collected, washed in 10 mL of Tyrode buffer and centrifuged again. This procedure was repeated twice [11]. The cells were then suspended in the concentration of 1 × 10^6 ^cells/mL in 10 mM HEPES-Tyrode buffer (pH 7.4) containing 0.1% BSA.

The 200 mL peritoneal cell suspensions were incubated with various concentrations of RCM-101 for 10 minutes at 37°C and then exposed to compound 48/80 for 10 minutes. Aliquots of 100 μL of rat peritoneal mast cells in Tyrode buffer were combined with 100 μL aliquots of RCM-101 extract in Tyrode buffer such that 5 × 10^5 ^cells/mL were incubated with RCM-101 at concentrations of 1, 10 and 100 μg/mL for 10 minutes immediately prior to stimulation of the cells with compound 48/80. The cell suspensions were then centrifuged at 4°C at 4800 rpm and the supernatant collected. As an internal standard, 10 μL of spermidine (1 mg/mL) was added to 200 mL aliquots of the supernatant, followed by 20 μL of 30% perchloric acid (HCIO_4_). The mixture was then filtered and 100 μL was transferred into HPLC vials for histamine determination [11]. The Ca^2+ ^chelating agent, EDTA, (100 μM) was used as a positive control.

The HPLC system (Shimadzu, Kyoto, Japan), which included a fluorescent detector (Shimadzu RF10XL), C-10ATvp pumps, SIL-10ADvp auto-injector and STRODS-II reversed phase column, equipped with post-column derivatisation was set up as previously described [11]. Samples of the peritoneal cell supernatant/internal standard solution were injected into the HPLC system using an autosampler. Histamine and spermidine were detected with excitation and emission wavelengths of 360 nm and 440 nm, respectively. Four-point standard curves for histamine were prepared, ranging from 50 – 2500 ng/mL in 10 mM HEPES-Tyrode buffer.

### Leukotriene B_4 _production

Synthesis of LTB_4 _was induced in neutrophils as previously described [15], with slight modification. Porcine blood was collected from a local abattoir. Neutrophils were isolated using a Percoll gradient and suspended in Hanks' buffer, containing 5 mM HEPES. Suspended neutrophils (2.8 × 10^6 ^cells/mL) were incubated (37°C) with RCM-101 extract, NDGA (as a positive control, 0.1, 1 or 10 μg/mL) or vehicle (ethanol), for 5 minutes before the addition of arachidonic acid (2.5 μM) substrate. Porcine neutrophils were suspended in Hanks' buffer in concentration of 2.8 × 10^6 ^cells/mL. RCM-101 (0.1, 1, 100 μg/mL) was added 10 minutes before the calcium ionophore A23187 (2.5 μM). After 5 minute incubation, production of LTB_4 _was initiated by the addition of the calcium ionophore A23187 (2.5 μM) and 5 minutes later the reaction was terminated by adjusting the pH to 3 with citric acid. PGB_2 _(45 ng) and 15-HETE (83 ng) were then added as internal standards. The reaction mixture was extracted with 5 mL of chloroform/methanol (7:3 v/v) and dried under vacuum. The residue was dissolved in 120 μL of HPLC mobile phase (methanol-water-acetic acid, 76/34/0.08, v/v/v, pH 3.0) and leukotriene metabolites were assayed using a Waters HPLC system equipped with an auto sampler, a multi-solvent delivery system and a Waters 996 Photodiode Array Detector. Standard curves were prepared by the addition of LTB_4 _(10 – 200 ng) and 5-HETE (500 – 800 ng) to neutrophil suspensions. Data were analysed using Water Millenium Software, Version 3.2, results being expressed as percentage of the vehicle control which was taken as 100%.

### Prostaglandin E_2 _production

Murine macrophages (Raw 264.7 cells, American Type Culture Collection, Rockville, MD, USA) were grown in RPMI 1640 medium, supplemented with 10% heat-inactivated FBS, 100 μg/mL gentamycin, 1.5 g/L sodium bicarbonate and 10 mM HEPES, at 37°C, in an atmosphere containing 5% CO_2_. Cells were sub-cultured once a week by harvesting them with trypsin/EDTA and seeding them in 75 cm^2 ^flasks. Once confluent, murine macrophages were suspended in serum-free RPMI medium at concentration of 2 × 10^5 ^cells/mL, and the cells were seeded in 24-well plates (1 × 10^5 ^cells/well), in serum-free RPMI medium. Cells were then treated with RCM-101 (1, 10, or 100 μg/mL) or vehicle 10 minutes before the addition of LPS (1 μg/mL). The supernatant and the cells were separated. PGE_2 _was assayed in the supernatant using an immune-enzyme analysis kit. The assay depends on competition between PGE_2 _and PGE_2_acetylcholinesterase conjugate (PGE_2_-tracer) for a limited amount of monoclonal PGE_2_antibody. The assays were carried out according to the manufacturer's protocol, in triplicate. PGE_2 _release was calculated using software supplied by the kit manufacturer.

### Determination of COX-1 and COX-2 protein expression in Raw 264.7 cells

Cultured Raw 264.7 cells prepared as described above for determination of PGE_2 _production, with and without incubation with RCM-101, were washed twice with ice-cold phosphate buffer saline then lysed with 100 μL/well of lysis buffer (50 mM Tris base, pH 7.6, 2 mM MgCl_2_, 1 mM EGTA, 1% TritonX, 1 mM phenyl PMSF, 1 mM pepstatin, 1 mM aprotinin, 1 mM leupeptin) for 5 minutes. The cells and the supernatant were collected and centrifuged for 5 minutes at 14000 rpm. The cell debris was discarded and the supernatant was assayed for protein concentration using Coomassie Protein Assay Kit (Bio-Rad Laboratories Pty Ltd, California, USA) and the UV-visible spectrophotometer (Cintra 5, GBC Scientific Equipment Pty Ltd, Illinois, USA)

COX-1 and COX-2 protein was measured by Western blotting as previously described [16] with a slight modification. Aliquots of 20 μg of total protein were loaded to each lane of 7.5% SDS-polyacrylamide gels. The proteins were then electrically transferred to nitrocellulose membranes which were incubated overnight with a polyclonal anti-rabbit COX-1 antibody or a monoclonal mouse anti-rat COX-2 antibody (diluted 1:500 and 1:2500 respectively) in 5% non-fat milk in Tris-buffered saline (Tris base 25 mM, glycine 19 mM, methanol 20%). On the next day, membranes were washed with Tris-buffered saline (Tris base 20 mM, NaCl 137 mM, Tween-20 0.1%, pH 7.5) for 40 minutes with constant agitation, during which time the buffer was changed every 5 minutes. The membranes were then incubated with swine anti-rabbit or goat anti-mouse secondary conjugated to horseradish peroxidase diluted 1:5000 with blocking buffer (Tris base 20 mM, NaCl 137 mM, Tween-20 0.1%, pH 7.5 and 5% non-fat milk). The results were visualized by enhanced chemiluminescence (Amersham Pharmacia Biotech) according to the manufacturer's instructions.

### Statistical analysis

Data are expressed as means ± standard deviation (SD). The statistical significance of differences between means was determined by unpaired, two-tailed Student's t-test or, for more than two groups, by first testing for global differences by one- or two-way analysis of variance (ANOVA) and then testing for differences between predetermined pairs of means by Dunnet's test. The differences with probability levels less than 0.05 (P < 0.05) were considered to be statistically significant.

## Results

### Inhibition of compound 48/80-induced histamine release from rat peritoneal mast cells

**Figure 1:**
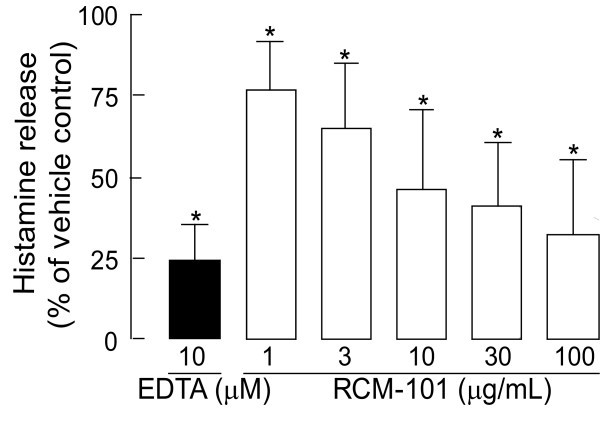
Inhibition of compound 48/80-stimulated histamine release from rat peritoneal mast cells by RCM-101. EDTA was used as a positive control. Data are plotted as means ± SD, (n = 8). *P < 0.05, One-way ANOVA and Dunnet's test

The amount of histamine released from rat unstimulated peritoneal mast cell preparations was 46.3 ± 37.1 ng/mL (n = 14). When the cells were stimulated with compound 48/80 (1 μg/mL) histamine release increased markedly to 638.3 ± 308 ng/mL (n = 14). As shown in Figure 1, compound 48/80-induced histamine release was inhibited by RCM-101 (1 – 100 μg/mL) in a concentration dependent manner. Compound 48/80-induced histamine release was also reduced by 10μM EDTA (Figure 1).

### Inhibition of leukotriene B_4 _production in porcine neutrophils

In the absence of calcium ionophore A23187 incubation, the production of LTB_4 _by porcine neutrophils was 9.58 ± 4.6 ng/mL. In vehicle (ethanol) control experiments, A23187 incubation increased LTB_4 _production to 167.77 ± 70.4 ng/mL (n = 8).

**Figure 2:**
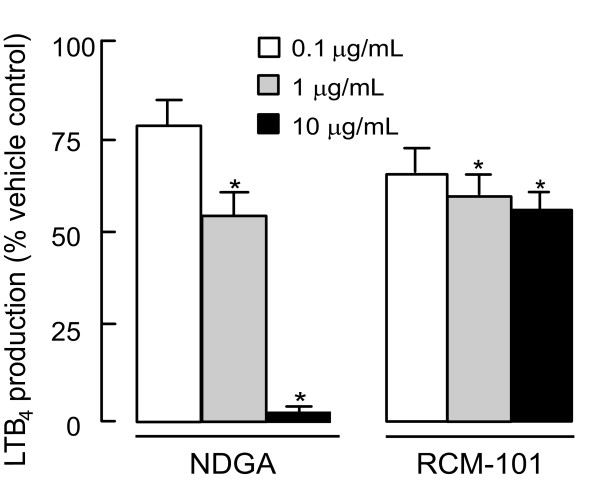
Inhibition of LTB_4 _formation in porcine neutrophils by RCM-101 and nordihydroguaiaretic acid (NDGA). Data are plotted as means ± SD, (n = 8 in each case). *P < 0.05, One-way ANOVA and Dunnet's test

As shown in Figure 2, LTB_4 _production in A23187-incubated neutrophils was inhibited by RCM-101 at concentrations of 1 and 10 μg/mL. NDGA (1 and 10 μg/mL) also inhibited A23187-induced LTB_4 _production.

### Inhibition of prostaglandin E_2 _production in LPS-stimulated Raw 264.7 cells

**Figure 3:**
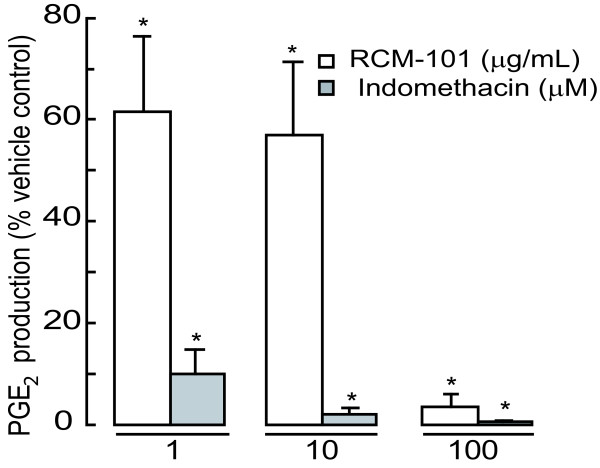
Inhibition of PGE_2 _production in LPS-stimulated Raw 264.7 cells by RCM-101 and indomethacin. Data are plotted as means ± SD of percentage control PGE_2 _production (n = 6 in each case). *P < 0.05, One-way ANOVA and Dunnet's test

Unstimulated Raw 267.4 cells incubated in serum-free RPMI medium for 24 hours produced a baseline concentration of PGE_2 _of 52 ± 24.4 pg/mL (n = 6). Incubating the cells with LPS (1 μg/mL) increased the PGE_2 _level to 3874 ± 818.13 pg/mL (n = 6). This induced production of PGE_2 _was reduced in a concentration-dependent manner by RCM-101 (1 – 100 μg/mL), when present during incubation with LPS. Indomethacin (1, 10 and 100 μM), when present during LPS incubation, completely blocked PGE_2 _production. The data are shown in Figure 3.

### Effects of RCM-101 on COX-1 and COX-2 protein expression in LPS-stimulated Raw 267.4 cells

**Figure 4:**
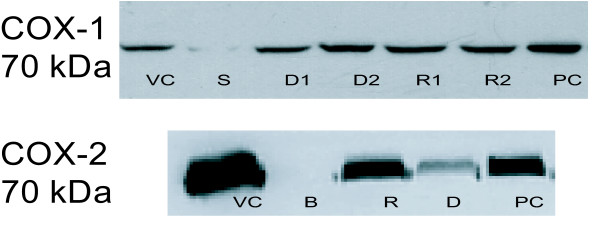
Western blot assay of COX-1 and COX-2 expression by macrophage (Raw 264.7. 20) cells after LPS stimulation. For each lane of SDS polyacrylamide gels, 20 μg of protein was loaded. COX-1 and COX-2. The proteins were detected on nitrocellulose membranes using specific antibodies and visualized by enhanced chemiluminescence. (A) **COX-1**: VC = vehicle control; S = separation lane (not loaded with protein); D1 = dexamethasone (100 μM); D2 = dexamethasone (10 μM); R1 = RCM-101 (100 μg/mL); R2 = RCM-101 (10 μg/mL); PC = positive control (COX-1 lysate). (B) **COX-2**: VC = vehicle control; B = cells not stimulated with LPS; R = RCM-101 (100 μg/mL); D = dexamethasone (100 μM); PC = positive control (COX-2 lysate).

As shown in Figure 4, immunoreactivity bands corresponding to COX-1 and COX-2 (70 kDa) were detected by Western blot analysis of the supernatant of lysed Raw 264.7 cells. Densiometeric analysis of the marker chemiluminescence indicated that expression of COX-1 protein was unaffected by RCM-101 (10 and 100 μg/mL). Similarly, dexamethasone (10 and 100 μM) also did not alter COX-1 protein expression. In contrast, the expression of COX-2 protein was significantly (P < 0.05, one-way ANOVA, Dunnet's test) reduced by 100 μg/mL RCM-101 and also by 100 μM dexamethasone. Figure 4 shows examples of the visualised bands on nitrocellulose membranes corresponding to COX-1 and COX-2 proteins.

## Discussion

This study was undertaken to extend our previous investigation of possible pharmacological mechanisms for the effects of the herbal formula RCM-101 in reducing SAR symptoms [10]. The main findings of the present study are that RCM-101 inhibits compound 48/80-induced release of histamine from isolated rat peritoneal mast cells and inhibits the production of LTB_4 _by porcine neutrophils and of PGE_2 _by Raw 264.7 cells. Histamine, PGE_2 _and LTB_4 _are well known mediators of inflammatory/allergic responses. Taken together with our previous findings in isolated tissues from rats and guinea-pigs, it seems that RCM-101, a herbal formula with 18 constituent Chinese herbs, has activity directed to inhibition of the synthesis or release of multiple key inflammatory mediators.

Mast cell-derived mediators, particularly histamine are considered to be responsible for the acute (early stage) allergic symptoms of SAR [17]. These mediators act on the smooth muscle cells of small blood vessels, blood platelets, mucous glands and sensory nerve endings to produce or contribute to symptoms such as nasal congestion, nasal and throat itching, sneezing and hypersecretion of mucus [18]. The release of mast cell-derived histamine is inhibited by RCM-101. While the inhibition mechanisms are not clear, RCM-101 has been shown to contain several herbal ingredients that inhibit the release or action of histamine. For example, *Rhizoma Cimicifugae *was reported to exert a potent inhibitory action on histamine-mediated contractions in guinea pig ileum [19] and *Flos Magnoliae *inhibits mast cell-mediated allergic reactions by preventing mast cell degranulation and IgE-mediated histamine release [20]. *Herba Schizonepetae *was also reported to reduce compound 48/80-induced histamine release [21]. Moreover, the Chinese herbal formula *Xiao Chai Hu Tang*, which contains several of the herbal ingredients of RCM-101, has also been shown to inhibit histamine release from rat peritoneal mast cells [11].

Limited information is available about the chemical constituents of the herbs in RCM-101 responsible for inhibition of the release or action of histamine or their action mechanisms. However, glycyrrhetinic acid, which is present in *Radix Glycyrrhizae*, a herbal component of RCM-101, was shown to inhibit the release of histamine by targeting protein kinase C-β (nPKC β) [22]. Conjugated linoleic acid, identified in *Rhizoma Chuanxiong*, another herbal component of the formula, is known to inhibit immediate anaphylaxis, histamine release and the synthesis of arachidonic acid metabolites [23].

Prostaglandins and leukotrienes were found to be involved in the pathophysiology of SAR [24] and LTB_4 _is released by infiltrating neutrophils during the immediate phase of allergic responses [25]. The present study found that RCM-101 inhibits the production/release of LTB_4 _induced by the calcium ionophore A23187 in porcine neutrophils. Previous studies showed that extracts of the herb *Radix Glycyrrhizae *inhibit A23187-induced release of arachidonic acid from cell membranes by inhibiting phospolipaseA_2 _and that they also inhibit 5-lipoxygenase, acting together to suppress the production of LTC_4 _and LTB_4 _[26]. Glycyrrhetinic acid and caffeic acid, present in *Radix Glycyrrhizae*, *Herba Menthae*, *Rhizoma Ligusticum Chuanxiong *and *Rhizoma Cimicifugae *[27], both were shown to inhibit arachidonic metabolite formation [28,29]. These findings suggest a possible action of RCM-101 on SAR through the inhibition of the release of LTB_4_.

PGE_2 _is released in both the early phase (from mast cells) and late phase (from basophils and eosinophils) responses of SAR [17]. We found that LPS-induced production of PGE_2 _by murine macrophages was inhibited by RCM-101. The findings are consistent with previous studies on individual herbal components of RCM-101. There is also evidence indicating that *Radix Glycyrrhizae *inhibits PGE_2 _production in rats tissues and *Rhizoma Cimicifugae *blocks LPS-induced production of PGE_2 _[19]. In addition, both topical and oral administration glycyrrhetinic acid was reported to prevent ear oedema and to inhibit PGE_2 _and LTC_4 _formation induced by arachidonic acid in mice [30]. These findings suggest a possible action of RCM-101 on SAR through the inhibition of PGE_2 _production.

The inhibition of prostaglandin production by RCM-101 is most likely due to inhibition of COX-2 protein expression, because we observed that COX-2 protein expression was markedly reduced by RCM-101 whereas the expression of COX-1 protein was unaffected. It is known that COX-2 is responsible for prostaglandin production in Raw 264.7 cells [14]. Previous studies also observed that *Rhizoma Cimicifugae *and *Radix Glycyrrhizae *inhibited COX-2 activity [26].

## Conclusion

The results obtained in this study indicate that RCM-101 has inhibitory actions on multiple inflammatory mediators, including the release of histamine from mast cells, and production of LTB_4 _and PGE_2 _by neutrophils and Raw 264.7 cells, respectively. In addition, RCM-101 also selectively inhibits the expression of the inducible enzyme COX-2. These actions of RCM-101 may contribute to its efficacy in SAR. The exact mechanisms of these actions and the contributions by individual herbal ingredients of RCM-101 require further investigation.

## Abbreviations

5-HETE: 5-hydroxyeicosatetraenoic acid

15-HETE: 15-hydroxyeicosatetraenoic acid

ANOVA: Analysis of variance

BSA: Bovine serum albumin

COX-1: Cyclooxygenase-1

COX-2: Cyclooxygenase-2

EDTA: Disodium ethylenediaminetetraacetic acid

EGTA: Ethylene glycol tetraacetic acid

HPLC: High performance liquid chromatography

IgE: Immunoglobulin E

LPS: Lipopolysaccharide

LTB_4_: Leukotriene B_4_

LTC_4_: Leukotriene C_4_

NDGA: Nordihydroguaiaretic acid

PGE_2_: Prostaglandin E_2_

PMSF: Phenylmethanesulphonylfluoride

SAR: Seasonal allergic rhinitis

SD: Standard deviation

## Competing interests

The author(s) declare that they have no competing interests.

## Authors' contributions

GBL conducted the experiments, contributed to the interpretation of the findings and preparation of the manuscript. CCLX contributed to the design of the study, the interpretation of the findings and preparation of the manuscript. DFS contributed to the interpretation of the findings and critically revised the manuscript. FCKT contributed to the design of the study and preparation of the manuscript. SM conducted some of the experiments, contributed to the interpretation of the findings and preparation of the manuscript. CGL contributed to the design and conduct of the study, the interpretation of the findings and preparation of the manuscript. All authors approved the final manuscript.
